# Supplementary material for: Doing Everything We Can to Help Our High-Risk Newborns: A Qualitative, Lifeworld-Led Study of What Early Risk Assessment for Cerebral Palsy Means to Parents
Source: J Clin Med. 2025 Apr 16;14(8):2740. doi: 10.3390/jcm14082740 (PMC12027544; doi:10.3390/jcm14082740)
Supplement: Supplementary file 1 [file jcm-14-02740-s001.zip › Table S3.pdf]

**HOW EARLY PREDICTION APPEARS TO PARENTS**	**A perspective framed by trauma and insecurity**		
	In the context of trauma, new parenthood and awareness of risk<br>Desire to know the child will live and be well<br>Any examination which may unveil signs of future disability or illness<br>Repeated assessments over time		
**ESSENTIAL STRUCTURES OF MEANING**	**On a spectrum from death to insignificancies**	**Living with uncertainty of what the parental role will entail**	**Seeing your child through the eyes of professionals, just in case**
	Grading worries<br>Relative to many things<br>Death overshadows all<br>CP can be mild or severe<br>Insignificancies remain	Preparing for the future<br>Parent-child bond<br>Worrying, exhaustion<br>Loneliness<br>Burden lifts gradually	Want of normalcy<br>Looking for signs<br>Objectification<br>Trusting HCP<br>Creating a safety net
**VARIATIONS BY RESULTS OF EARLY PREDICTIONS**	**How to move on after early predictions**		
	Low-risk after predictive assessments: Relieves the burden<br>Facilitates moving on<br><br>Uncertain after predictive assessments: Amplifies and prolongs the burden<br>Real yet elusive threat to the future		
