# Supplementary material for: Doing Everything We Can to Help Our High-Risk Newborns: A Qualitative, Lifeworld-Led Study of What Early Risk Assessment for Cerebral Palsy Means to Parents
Source: J Clin Med. 2025 Apr 16;14(8):2740. doi: 10.3390/jcm14082740 (PMC12027544; doi:10.3390/jcm14082740)
Supplement: Supplementary file 1 [file jcm-14-02740-s001.zip › Table S1.pdf]

MAIN QUESTIONS	FOLLOW-UP QUESTIONS
**Can you tell me about your experience with the GMA-assessment?**	Making the video?
	The technical aspects?
	Feel secure the recordings were correct and made it to the correct destination?
	Did you feel well cared for?
**What was it like to get the result of the GMA assessment?**	The waiting period?
	How did you get the message?
	What does the result mean?
	Do you believe in the result?
	Has the result had any consequences?
**What are your thoughts about the future?**	For your family?
	For your child?
