# Supplementary material for: Doing Everything We Can to Help Our High-Risk Newborns: A Qualitative, Lifeworld-Led Study of What Early Risk Assessment for Cerebral Palsy Means to Parents
Source: J Clin Med. 2025 Apr 16;14(8):2740. doi: 10.3390/jcm14082740 (PMC12027544; doi:10.3390/jcm14082740)
Supplement: Supplementary file 1 [file jcm-14-02740-s001.zip › Table S2.pdf]

OPEN APPROACH	ENCOURAGEMENT, GOING DEEPER, SPECIFYING LIVED EXPERIENCES
1: OPENING<br><br>**What is a normal day like for you?**	That sounds____ .
2: DIRECTINIG QUESTIONS<br><br>**So how do you relate to the risk, which is there, of your child having a lasing injury?**<br><br>**What was it like doing the GMA examination at three months age to assess the risk of CP?**	Can you tell me more about that?<br><br>In what way?<br><br>Do you have any examples?<br><br>What was that like?<br><br>What did you think/do in that moment?<br><br>What did that feel like?
